# Effects of 45° prone position ventilation in the treatment of acute respiratory distress syndrome: A protocol for a randomized controlled trial study

**DOI:** 10.1097/MD.0000000000025897

**Published:** 2021-05-14

**Authors:** Zhenye Zhan, Hairong Cai, Huiling Cai, Xingmin Liang, Shikeng Lai, Yajie Luo

**Affiliations:** a Guangzhou Panyu Central Hospital; b The Second Clinical Medical School, Guangzhou University of Chinese Medicine; c The Second Affiliated Hospital of Guangzhou University of traditional Chinese Medicine, Guangzhou, Guangdong Province, China.

**Keywords:** 45°, acute respiratory distress syndrome, clinical efficacy, prone position ventilation, protocol

## Abstract

**Background:**

Acute respiratory distress syndrome (ARDS) is an increasingly common acute respiratory failure that seriously threaten people's health. ARDS has a case fatality rate of up to 40%. ARDS is a serious threat to the life safety of patients and the quality of life, causing a huge economic burden to individuals, families and society. ARDS has become a large worldwide public health problem. Prone position ventilation (PPV) is an important auxiliary treatment for ARDS, which could improve oxygenation. However, PPV could cause Pressure injuries (PI) and other complications easily. We found that 45° PPV could reduce the incidence of PI, but lack of robust Evidence-based medicine evidence proving its efficacy. Therefore, we designed a randomized controlled trial to evaluate the efficacy of 45° PPV in the treatment of ARDS.

**Methods:**

A total of 268 patients will be randomly assigned to the control group and the test group (n = 134 each) in a ratio of 1:1. The treatment period is 7 days. The primary outcome measure will be the incidence of PI. The secondary outcomes will include APACHE II score, Braden Scale score, heart rate, systolic blood pressure, diastolic blood pressure, central venous pressure, mean arterial pressure, pH of arterial blood, oxygenation index, oxygen partial pressure, and carbon dioxide partial pressure. The evaluation will be performed at baseline, 1 hour, 12 hour, 48 hour, 5days, 7days after PPPV.

**Results:**

This study is helpful to evaluate the efficacy of 45° PPV in the treatment of ARDS.

**Conclusion:**

45° PPV may reduce the incidence of PI and improve oxygenation in patients with ARDS, which has important value in practical application

**Trial registration:**

ChiCTR2000040436, registration time: November 28, 2020.

## 1 Introduction

Acute respiratory distress syndrome (ARDS) is an increasingly common acute respiratory failure that seriously threaten people's health. ARDS has a case fatality rate of up to 40%.^[1,2]^ The concept of adult respiratory distress syndrome was first proposed by Ashbaugh et al in 1967.^[3]^ With the continuous understanding of ARDS, it was found that ARDS occurs not only in adults but also in children, The American Thoracic Society (ATS) and European Society of Intensive Care Medicine have renamed adult respiratory distress syndrome as ARDS.^[4,5]^

ARDS is a clinical syndrome characterized by acute, progressive dyspnea and intractable hypoxemia, which is caused by various intrapulmonary and/or extrapulmonary causes other than cardiogenic factors. ARDS is an acute respiratory failure caused by diffuse alveolar and interstitial edema following the damage to pulmonary capillary endothelial cells and alveolar epithelial cells. ARDS is characterized by progressive dyspnea and intractable hypoxemia, non-cardiogenic pulmonary edema, opacity of bilateral chest X-ray films and decreased lung compliance. The etiology of ARDS includes shock, severe infection, trauma, severe burn, pancreatitis, pneumonia, drug overdose and poisoning, blood transfusion, etc.^[6–9]^

Piehl et al^[10]^ first reported the efficacy of prone position ventilation (PPV) in ARDS, and he found that PPV can improve oxygenation by recruiting alveoli situated in dorsal dependent regions, which is consistent with the results of subsequent clinical studies.^[11–13]^ PPV could improve the pulmonary ventilation/blood flow ratio, oxygenation and reduce the mortality by recruiting alveoli situated in dorsal dependent regions and promoting the redistribution of lung gas.^[14–17]^ One meta-analyses have shown that PPV could reduce mortality in patients with severe ARDS.^[12]^ Gueirn et al.^[15]^ found that early application of PPV can significantly reduce the 28-day and 90-day mortality rate mortality in patients with severe ARDS. More than 70% of lung injury patients could clinically improve oxygenation by using PPV.^[18]^

However, a clinical application of PPV is difficult especially for critically ill patients with various pipelines and obese patients. Even worse, PPV could cause a variety of complications, such as hemodynamic instability, pressure injuries (PI), aspiration, conjunctival edema, as well as the compression, displacement and prolapse of various indwelling catheters caused by postural changes, among which the PI is the most common. Manzano et al.^[19]^ found that the incidence of PI in patients with mechanical ventilation for more than 7 days was as high as 35.6%. Mechanical ventilation time was a risk factor for PI in critically ill patients.^[20,21]^ Sud S et al^[22]^ found that the incidence of PI was 42.7% in patients with ARDS using PPV.

We found that 45 °PPV could reduce the incidence of PI, but lack of robust Evidence-based medicine evidence proving its efficacy. Therefore, we aim to conduct a randomized controlled clinical trial (RCT) to evaluate the efficacy of 45 ° PPV in the treatment of ARDS.

## 2 Methods

### 2.1 Trial design and registration

**Figure 1:**
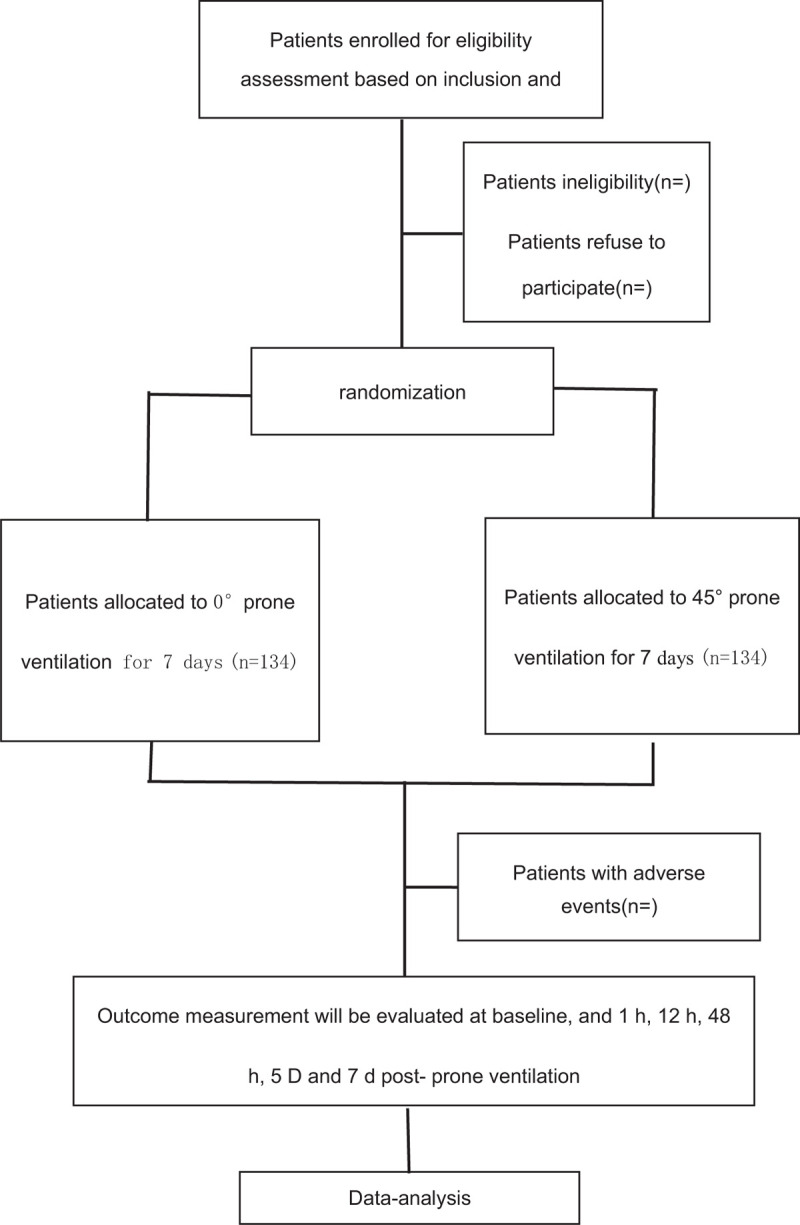
Study flow diagram.

**Figure 2:**
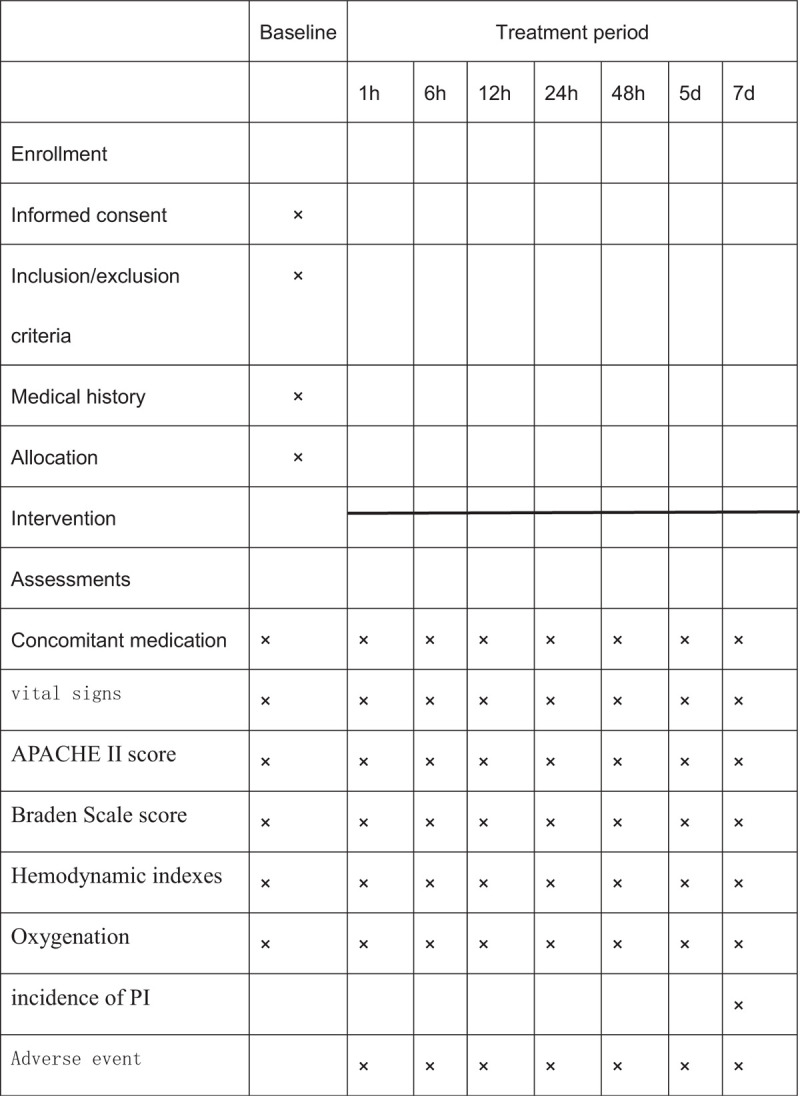
Study design schedule.

This is a randomized controlled clinical trial of 45° PPV (test group) compared to 0° PPV (control group). Participants will be recruited from the Department of Emergency and Intensive Care Medicine, Guangzhou Panyu central hospital. The Figure 1 shows the study design in the flowchart, and the Figure 2 illustrates the time schedule of enrolment, interventions, assessments, and visits of participants. We developed the protocol based on the Standard Protocol Items: Recommendations for Intervention Trials guidelines (Additional file 1).

This protocol has been registered in the Chinese Clinical Trial Registry on November 28,2020, http://www.chictr.org.cn/showprojen.aspx?proj=64955. And the registration number is ChiCTR2000040436.

### 2.2 Patient population and eligibility criteria

#### 2.2.1 Inclusion criteria

(1) Patients meet the Berlin diagnostic criteria for ARDS^[23]^;
(2) Patients with ARDS require invasive mechanical ventilation;
(3) The onset time of ARDS is less than 72 hours;
(4) The age ranged from 18 to 75 years old;
(5) Those who agree to participate in this study and are willing to sign informed consent.

#### 2.2.2 Exclusion criteria

(1) Under 18 years old or over 75 years old;
(2) Pregnant or breastfeeding women;
(3) Patients with PI at admission;
(4) Patients with contraindications for PPV including intracranial hypertension, recent maxillofacial/thoracic surgery, massive hemoptysis, spinal/pelvic instability, mean arterial pressure  < 65mm Hg;
(5) Hemodynamic instability;
(6) Severe pneumothorax;
(7) The survival time is expected to be lower than 24 hours;
(8) Previous or current confirmed diagnosis of pulmonary bullae, spontaneous pneumothorax and Chronic obstructive pulmonary disease;
(9) There is active bleeding in the respiratory tract.

### 2.3 Sample size

The sample size was calculated based on the incidence of 7-day PI. According to the previous literature,^[24]^ we hypothesized that the incidence of 7-day PI in the control group and the test group will be 42.7% and 22.7%, respectively. Set ɑ = 0.05 and □ = 0.10. We use PASS 15 software (PASS NCSs, LLC, USA) to calculate the size of the control group and test group, N1 = N2 = 111. Assuming that 20% of patients may be lost during follow-up, a total of 268 patients will be recruited.

### 2.4 Randomization

Patients will be randomly allocated into either to the control group and the test group according to the ratio of 1:1. The random allocation sequence listing will be generated by SPSS software operated by a third party who will not participate in the clinical study. The randomization information will be concealed until the end of the trial.

### 2.5 Blinding

Because of the nature of PPV, doctors and patients will know the grouping information. However, investigator, statisticians, and outcome evaluators will be blinded to group allocation.

### 2.6 Researchers

Five investigator will be responsible for data collection. Two supervisors were responsible for monitoring the whole experiment.

### 2.7 Recruitment

Patients will be recruited in the department of Emergency and ICU, Guangzhou Panyu central hospital. The poster will briefly provide the trial and treatment details, as well as contact information. Interested participants will be able to contact the researchers directly. We will provide potential participants with detailed information describing the benefits and possible risks of the trial. If they decide to participate, they will ask sign written informed consent informed consent. We will strengthen doctor-patient communication to improve the compliance.

### 2.8 Patient safety

The adverse events associated with PPV included severe hypoxemia, pneumothorax and intracranial hypertension. Clinicians will record the occurrence of any adverse events and the intervention will be stopped immediately in case of serious adverse event. The independent safety supervision committee composed of three experts from different fields in Guangzhou Panyu central hospital has the right to terminate the study.

## 3 Interventions

### 3.1 Basic treatment

All patients will be endotracheally intubated and mechanically ventilated with tidal volume 6 ml/kg (ideal body weight), inspiratory to expiratory ratio 1:2–1:3, respiratory rate less than 20 times/min. And PEEP and FiO2 will be adjusted according to the ARDSnet protocol.

### 3.2 The control group

The patients in the control will undergo mechanical ventilation in the 0°prone position for 16 hours a day and turning over every 2 hours. we will remove the electrodes in the anterior chest area and paste hydrocolloid dressings on the clavicle and the anterior superior iliac spine. The electrode will be sticked on the back.

### 3.3 The test group

The patients in the test group will undergo mechanical ventilation in the 0°prone position for 2 hours, then tilted 45° towards prone to the left for 2 hours and tilted 45° towards prone to the right for 2 hours, and returned to 0 ° prone position finally. This cycle will last for 16 hours.

### 3.4 The course of treatment

The duration of treatment will be 7 days.

#### 3.4.1 Combined treatment regulations

During study, it is forbidden to use PPV in other positions.

### 3.5 Outcome measures

#### 3.5.1 Primary outcome

The primary outcome will be the incidence of PI. Outcome indicators will be collected at day 7 of PPV.

#### 3.5.2 Secondary outcomes

(1) APACHE II score: APACHE-II score includes 12 physiological scores, 1 age score, 1 Glascow scores and 5 chronic disease scores. The APACHE II score ranged from 0 to 71 points and a higher score indicated a worse severity. The APACHE II score will be evaluated at baseline, and 1 hour, 12 hour, 48 hour, 5 days and 7 days post-PI.
(2) Braden Scale score: The Braden Scale score has been widely used for predicting PI risk. The Braden Scale score ranged from 6 to 23 points and a higher score indicated a high PI risk. The Braden Scale score will be evaluated at baseline, and 1 hour, 12 hour, 48 hour, 5 days, and 7 days post-PI.
(3) 3) Hemodynamic indexes: The hemodynamic indexes include HR, systolic blood pressure, diastolic blood pressure, central venous pressure, mean arterial pressure. The hemodynamic indexes will be evaluated at baseline, and 1  hour, 12  hour, 48  hour, 5 days, and 7 days post-PI.
(4) 4) Oxygenation: The Oxygenation include pH of arterial blood, oxygenation index, partial pressure of oxygen and partial pressure of carbon dioxide. The Oxygenation will be evaluated at baseline, and 1 hour, 12  hour, 48  hour, 5 days, and 7 days post-PI.

#### 3.5.3 Safety outcomes

Other complications of PPV include unplanned extubation, catastrophic hypoxemia, pneumothorax, eye complications and intracranial hypertension. If an adverse event (AE) occurs, the investigator will record the details (including symptoms, time of occurrence, duration, examination, and results) in the form of a case record form (CRF). Serious adverse event (SAE) will be reported to the ethics committee of Guangzhou Panyu Central Hospital, and relevant rescue procedures will be started immediately.

#### 3.5.4 Data management

Test data will be recorded in paper-based and electronic CRF. An independent data-monitoring committee reviewed the data every 6 months. Only investigator and monitors could access the electronic CRF. Clinicians and data analysts will not be able to access the data during the evaluation process. At the end of the trial, the database will be locked by the data management team, after which the investigator cannot modify the data. Paper and electronic documents will be kept for at least 5 years.

### 3.6 Ethics

This study has been approved by the ethics committee of Guangzhou Panyu Central Hospital (ethics No.: YF2020–104-13) and registered in China clinical trial registration center on November 26, 2020. The trial strictly followed the Helsinki Declaration (2000 EDITION). Only patients with informed consent can participate in the trial.

### 3.7 Dissemination

The results of this study will be disseminated through scientific conferences and per-papers. According to reasonable requirements, the readers could contact the corresponding author to obtain the data.

### 3.8 Confidentiality

All personal information of the participants will be kept strictly confidential and the anonymized individual patient data will be shared on request.

### 3.9 Ancillary and post-trial care

In case of injury related to this study, the subjects can obtain corresponding medical and nursing care. Meanwhile, they are entitled to compensation.

### 3.10 Statistical analysis

Statistical analysis will be performed on an intention-to-treat-basis. The measurement data will be prestend

In this trial, we will conduct intention to treat analysis and protocol analysis. The measurement data will be expressed as mean ± standard deviation and the enumeration data will be expressed as in percentage. The student *t*-test will be performed for measurement data and chi square test or Fisher exact probability methods for enumeration data. All data statistics were conducted using a bilateral test, and statistical significance was set at *P* < .05. The clinical results will be analyzed by SPSS 22.0 statistical software (IBM SPSS statistics, IBM Corp, Somers, NY).

## 4 Discussion

ARDS is characterized by severe and life-threatening acute respiratory syndrome with a high mortality. PPV is an important method to improve the alveolar ventilation in patients with ARDS. PPV can improve oxygenation in patients with ARDS by increasing alveolar ventilation, re-distributing transpulmonary pressure, opening alveoli, reducing the risk of alveolar hyperinflation, reducing the compression of mediastinum on lung tissue, improving the pulmonary ventilation/blood flow ratio, reducing intrapulmonary shunt, clearing the sputum from the trachea easily. However, there are complications in about such as hemodynamic instability, skin and mucous membrane PI, aspiration, conjunctival edema, and various indwelling catheter compression, displacement and prolapse caused by body position change, among which the PI is the most common. We found that 45 °PPV could reduce the incidence of PI, but lack of robust Evidence-based medicine evidence proving its efficacy. Therefore, we aim to conduct a RCT to evaluate the efficacy of 45 ° PPV in the treatment of ARDS.

RCT is the gold standard to evaluate the efficacy and safety of drugs. Of course, this study also has some shortcomings, including:

(1) Due to the serious condition of the subjects, the loss rate is high, which will affect the results of the study. Therefore, we should maintain a good relationship between doctors and patients in the research process;
(2) The patients in this study are limited to Chinese patients, so the results of this study have some limitations and may not be extended to other regions.

### 4.1 Trial status

Protocol version number V1.0, October 10.2020. The trial is currently in the recruitment phase of participants. Recruitment will be beginning on December 1, 2020 and is expected to end on December 31, 2023.

## Acknowledgments

We would like to thank all the patients who will participate in the trial and the staff for their support.

## Author contributions

**Conceptualization**: Hairong Cai, Huiling Cai.

**Data curation:** Hairong cai.

**Investigation**: Xingmin Liang.

**Resources:** Huiling Cai.

**Supervision**: Yajie Luo.

**Visualization:** Xingmin Liang.

**Writing – original draft**: Zhenye Zhan.

**Writing – review & editing**: Zhenye Zhan, Yajie Luo.
